# Seroprevalence of Toxoplasmosis in Sheep and Its Zoonotic Importance in Hargeisa, Somaliland

**DOI:** 10.1002/puh2.70035

**Published:** 2025-02-21

**Authors:** Abdiqani Mohamed Jama, Taddesse Yayeh Yihun

**Affiliations:** ^1^ College of Agriculture and Veterinary Medicine University of Hargeisa Hargeisa Somaliland; ^2^ School of Veterinary Medicine Bahir Dar University Bahir Dar Ethiopia

**Keywords:** Hargeisa, pregnant women, seroprevalence, sheep, *Toxoplasma gondii*

## Abstract

**Background:**

Toxoplasmosis has public health importance, particularly in immunocompromised individuals such as pregnant women leading to congenital abnormalities and fetal losses. In this study, we aimed to determine the seroprevalence of toxoplasmosis in sheep at Mandeeq Slaughter House in Hargeisa and pregnant women attending antenatal care at Hargeisa Group Hospital.

**Methods:**

Cross‐sectional study design with systematic random sampling method and Questionnaire surveys were used for the collection of data from sheep and pregnant women. Sera were collected and examined for anti‐*Toxoplasma gondii* antibodies using latex agglutination test.

**Results:**

The overall prevalences of toxoplasmosis were 25.5% and 28% in sheep and human, respectively. Multivariable logistic regression analysis indicated that female (AOR = 2.18; 95% CI: 1.38–3.47; *p* = 0.001) and young age groups of sheep (AOR = 3.04; 95% CI: 1.04–8.86; *p* = 0.041) were significantly associated with *T. gondii* seropositivity. In pregnant women, age groups between 25 and 34 (AOR = 2.76; 95% CI: 1.07–7.14; *p* = 0.037), pregnant women who have cats in their home (AOR = 6.45; 95% CI: 2.37–17.52; *p* = 0.000), women who have close contact with garden soil (AOR = 6.74; 95% CI: 2.55–17.81; *p* = 0.000), poor hand washing practices before food eating (AOR = 29.5; 95% CI: 5.41–161.11; *p* = 0.000), and drinking tap water (AOR = 8.4; 95% CI: 2.54–28.08; *p* = 0.000) were significantly associated with *T. gondii* seropositivity.

**Conclusion:**

Toxoplasmosis is prevalent in sheep and pregnant women in Hargeisa. We recommend that pregnant women should avoid eating uncooked mutton, reduce gardening activities, keep personal and environmental hygiene, and drink boiled water to reduce the risk of the toxoplasmosis.

## Graphical abstract

• Toxoplasmosis is highly prevalent in Hargeisa, Somaliland.

• Contaminated tap water and poor personal hygiene could be a source of toxoplasmosis for pregnant women in Hargeisa.

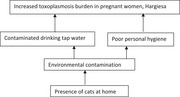

## 1 Introduction

Toxoplasmosis, caused by *Toxoplasma gondii*, is a zoonotic disease that commonly infects sheep [1] and the public with economic impacts everywhere [2]. It is an emerging foodborne parasitic disease affecting one‐third of the human population [3]. Toxoplasmosis is transmitted by ingestion of food, water, vegetables, and fruits contaminated with sporulated oocysts sheded from cats or ingesting tissue cysts from raw or undercooked meat; or transplacental transmission of tachyzoites [4]. In the case of immunocompromised pregnant women, toxoplasmosis is associated with severe neurological or ocular diseases such as chorioretinitis, blindness, myocarditis associated with various complications, cerebral anomalies, hydrocephalus, intracranial calcifications, microcephaly, convulsions, schizophrenia, and epilepsy [5, 6, 7, 8, 9].

East Africa is spotted as a high‐risk area for toxoplasmosis due to close linkage between human and livestock, sociocultural practices, poor environmental hygiene, and poverty [10]. In this region, more than one‐half of pregnant women were infected [11]. Despite the few studies on the high prevalence of human toxoplasmosis in Somalia [12], the status of toxoplasmosis both in animals and humans in Somaliland, the secessionist northwestern slice of Somalia that declared independence in 1991 [13], remained unknown. Therefore, this study aimed to estimate the seroprevalence of toxoplasmosis in sheep and pregnant women in Hargeisa, Somaliland.

## 2 Materials and Methods

### 2.1 Study Area

**FIGURE 1:**
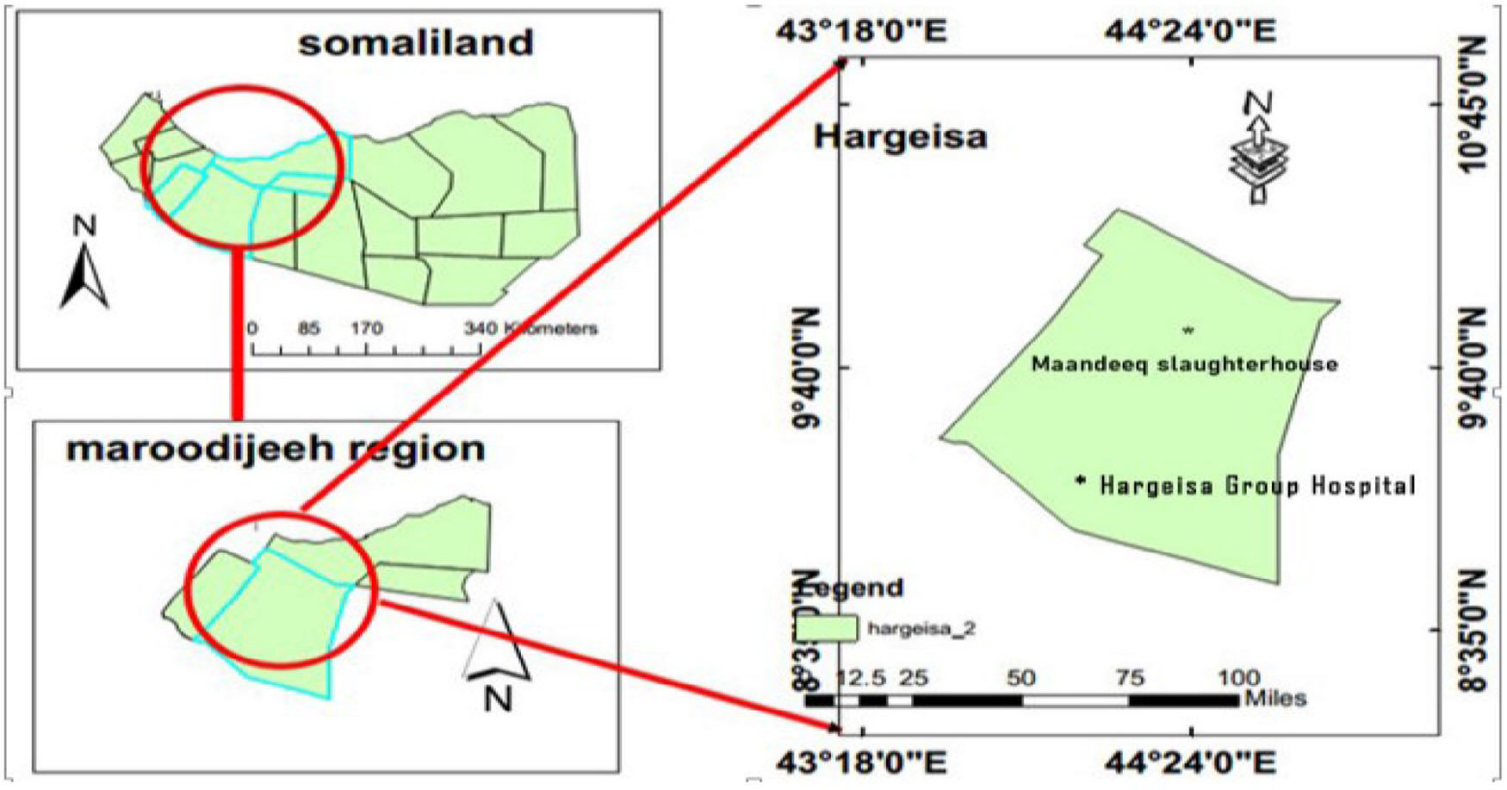
Map of Somaliland showing the location of study sites.

Hargeisa is the capital city of Somaliland. It is situated in the western part of Somaliland about 160 km Northeast of Jigjiga. It is considered to be the most populous city in Somaliland. The altitude of this city is around 1334 m above sea level, and it is located between 9.5624° N latitude and 44.0770° E longitude. Hargeisa, semi‐arid type of climate, receives the bulk of its precipitation between the months of April and September with an annual rainfall of 400 mm. The average monthly temperature of Hargeisa ranges from 18°C in December and January to 24°C in June. Data were collected from two locations of Hargeisa: *Mandeeq* abattoir and Hargeisa Group Hospital (HGH) (Figure 1).

### 2.2 Study Design

A cross‐sectional study was conducted to estimate the prevalence of toxoplasmosis and identify potential risk factors associated with seropositive pregnant women attending antenatal care (ANC) in HGH (referral public hospital) from June 2021 to December 2021.

### 2.3 Study Population, Inclusion, and Exclusion Criteria

The source population were sheep slaughtered at Mandeeq slaughter house for human consumption, and all pregnant women visiting HGH for ANC. Pregnant women 18 years old and above were considered during the study period. This study excluded those pregnant women refusing for participation and those who are sick at the time of attending ANC.

### 2.4 Sample Size and Sampling Method

An estimated number of 400 sheep were sampled. Blood collection was arranged to complete within 2 weeks. Thus, each day for a period of 2 weeks, 28 sheep were selected by systematic random sampling method. We also estimated to sample 200 pregnant women from HGH within a week. Each day, around 50 pregnant women had a visit to the Hospital, of which 28 of them were sampled daily.

### 2.5 Blood Sample Collection and Serum Preparation

Aseptically, around 5 mL of blood was drawn from the jugular vein of sheep and allowed to clot for serum separation. After consenting pregnant women, 5 mL of venous blood was collected by laboratory nurses into a plain vacutainer tube by using sterile disposable syringe. The tubes were appropriately labeled with the identity number of participants and the blood was allowed to coagulate at room temperature. All samples were sent to Somaliland National Veterinary Laboratory (SNVL), and serums were separated from the whole blood by centrifugation at 2800 × *g* for 5 min. Serum samples were aliquoted into two portions by using a sterile Pasteur pipette and stored at −20°C before performing serological tests for *T. gondii*.

### 2.6 Questionnaire Survey

Questionnaire survey was conducted to identify potential risk factors associated with the existence of toxoplasmosis in pregnant women. Factors such as location, age, pregnancy status, stage of pregnancy, presence of cats at home, physical contact with cats, and consumption of raw/undercooked meat, consumption of raw vegetables and fruits, and source of drinking water were considered.

### 2.7 Principle and Performance of the Test

Toxo‐latex test is a rapid slide agglutination procedure, developed to detect more than 10 IU/mL anti‐toxoplasma antibodies. The assay was performed by testing a suspension of latex particles coated with antigenic extract of *T. gondii* against unknown samples. As described by manufacturer, 50 µL sera samples and 25 µL of Toxo‐latex reagent were placed on the Toxo‐latex agglutination slide and one drop of each positive and negative control separate circles on the slide text and mixed thoroughly. The presence or absence of a visible agglutination indicates the presence or absence of anti‐toxoplasma antibodies in the sample.

### 2.8 Quality Control

Every day, the collected data were reviewed and checked for completeness. For laboratory investigations, standardized operating procedures and manufacturer's instructions were strictly followed. The quality of latex agglutination kits for anti‐*T. gondii* was checked by both positive and negative controls.

### 2.9 Data Management and Analysis

Data from questionnaire and laboratory findings were coded and entered into SPSS version 26.0 for descriptive statistics and multiple logistic regression analysis. A *p* value less than 0.05 were considered to be statistically significant.

## 3 Result

### 3.1 Prevalence of Toxoplasmosis and Its Associated Risk Factors in Sheep

A total of 400 serum samples were tested for *T. gondii*–specific antibodies. One hundred two sera were seropositive for *T. gondii* with an overall seroprevalence of 25.5% of all tested samples. In both univariable and multivariable logistic regression analyses, sex and age of sheep were risk factors associated with *T. gondii* seropositivity.

Multivariable logistic regression analysis showed that female sheep were two times more likely becoming *T. gondii* seropositive when compared to males ((AOR = 2.18; 95% CI: 1.38–3.47; *p* = 0.001)). Likewise, the young age group of sheep were three times more likely becoming *T. gondii* seropositive when compared to adult age groups (AOR = 3.04; 95% CI: 1.04–8.86; *p* = 0.041) as shown in Table 1.

**TABLE 1:** Multivariable logistic regression analysis of *Toxoplasma gondii* infection and potential risk factors associated with sheep slaughtered at Mandeeq slaughter house.

Variables	Number of examined (*N* = 400)	Number of positive % (*N* = 102)	COR 95% (CI)	*p* value	AOR 95% (CI)	*p* value
**Breed of animal**						
Local Non local	392 8	100 (25.5%) 2 (25%)	0.97 (0.19–4.90) Ref 1.00	0.974	0.92 (0.17–4.85) Ref 1.00	0.927
**Sex of animal**						
Female Male	225 175	42 (18.7%) 60 (34.2%)	2.27 (1.43–3.59) Ref 1.00	0.001	2.18 (1.38–3.47) Ref 1.00	0.001
**Age of animal**						
Young Adult	41 359	4 (9.8%) 98 (27.3%)	3.36 (1.16–9.70) Ref 1.00	0.041	3.04 (1.04–8.86) Ref (1.00)	0.041

Abbreviations: AOR = adjusted odds ratio; CI = confidence interval; COR = crude odds ratio.

### 3.2 Prevalence of Toxoplasmosis and Its Associated Risk Factors in Pregnant Women

Among 200 pregnant women tested for *T. gondii* antibodies, 56 were found to be seropositive with an overall prevalence of 28%. In multivariable logistic regression analysis, potential risk factors significantly associated with *T. gondii* seropositivity were age groups between 25 and 34, cat availability at home, close contact of pregnant women with cats, contact with garden soil, poor hand washing habit before eating, and drinking tap water were significantly associated with *T. gondii* seropositivity (Table 2).

**TABLE 2:** Multivariable logistic regression analysis of *Toxoplasma gondii* infection and potential risk factors associated with pregnant women.

Variables	Number of examined (*N*=200)	Number of positive % (*N* = 56)	COR 95% (CI)	*p* value	AOR 95% (CI)	*p* value
**Age**						
15–24	26	6 (23%)	2.41 (0.82–7.04)	0.107	1.35 (0.34–5.32)	0.534
25–34	124	29 (23.3%)	2.37 (1.17–4.77)	0.015	2.76 (1.07–7.14)	0.037
35–44	50	21 (42%)	Ref 1.00			
**Trimesters**						
First	36	7 (19.4%)	4.14 (1.41–12.17)	0.010	2.73 (0.67–4.05)	0.158
Second	132	33 (25%)	3.03 (1.35–6.65)	0.007	2.87 (0.96–9.11)	0.074
Third	32	16 (50%)	Ref 1.00			
**History of abortion**						
Yes	50	21 (42%)	2.37 (1.20–4.69)	0.012	0.67 (0.28–1.73)	0.415
No	150	35 (23.3%)	Ref 1.00			
**Having cat at home**						
Yes	30	18 (60.1%)	5.21 (2.30–11.70)	0.000	6.45 (2.37–17.52)	0.000
No	170	38 (22.3%)	Ref 1.00			
**Close contact with cat**						
Yes	33	25 (75.5%)	0.15 (0.06–0.33)	0.000	0.12 (0.04–0.35)	0.000
No	167	31 (18.4%)	Ref 1.00			
**Eating raw vegetables**						
Yes	89	25 (28%)	0.99 (0.53–1.84)	0.992	1.09 (0.45–2.62)	0.838
No	111	31 (27.9%)				
**Close contact with garden soil**						
Yes	32	19 (59.3%)	5.17 (2.33–11.45)	0.000	6.74 (2.55–17.81)	0.000
No	168	37 (22%)	Ref 1.00			
**No hand washing before eating**						
Yes	185	44 (23.7%)	12.81 (3.4–47.48)	0.000	29.5 (5.4–161.11)	0.000
No	15	12 (80%)	Ref 1.00			
**Source of drinking water**						
Tap water	136	48 (35.3%)	3.81 (1.68–8.67)	0.001	8.4 (2.54–28.08)	0.000
Well	64	8 (12.5%)	Ref 1.00			

Abbreviations: AOR = adjusted odds ratio; CI = confidence interval; COR = crude odds ratio.

After adjustment for possible confounder, adult age groups (25–34) of pregnant women were three times more likely to develop toxoplasmosis than younger age groups (AOR = 2.76; 95% CI: 1.07–7.14; *p* = 0.037). Pregnant women having cats in their home were six times more likely of becoming *T. gondii* seropositive than those who were not keeping cats in their home (AOR = 6.45; 95% CI: 2.37–17.52; *p* = 0.000). Pregnant women who had contact with garden soil were almost seven times more likely to become toxoplasmosis seropositive than those who did not have contact with garden soil (AOR = 6.74; 95% CI: 2.33–11.45; *p* = 0.000). Likewise, pregnant women who were unable to wash their hands frequently were 29 times more likely to acquire toxoplasmosis than those individuals washing their hands before eating (AOR = 29.5; 95% CI: 5.41–160.11; *p* = 0.000). We also found that pregnant women drinking tap water were eight times more likely to acquire *T. gondii* infection than those who use well water (AOR = 8.4; 95% CI: 2.54–28.08; *p* = 0.000) (Table 2).

## 4 Discussion

Toxoplasmosis is a widespread disease with high prevalence and public health importance. East Africa region is a high‐risk area of toxoplasmosis owing to a close association between humans and livestock, sociocultural practices, poor environmental hygiene, and poverty [14]. This study showed a seroprevalence 25.5% in sheep waiting for slaughter at Mandeeq slaughter house. This finding is consistent with the high prevalence of ovine toxoplasmosis in the nearby country, Somalia [15]. In this study, female and young sheep were significantly associated with seropositivity of toxoplasmosis. This could probably be related to reduced immunity owing to hormonal influences in females and reduced the exposure of lambs for oocysts [16].

Studies in Somalia also indicated that toxoplasmosis has been highly prevalent in human patients and pregnant women [12]. Our study in Hargeisa also indicated a high prevalence of toxoplasmosis (28%) in pregnant women who were attending ANC in Hargeisa Group Hospital, indicating that this disease could be endemic in the study area. We also observed a significant association of toxoplasmosis seropositivity with age category. Pregnant women who were in the age group 25–34 were more likely to develop toxoplasmosis than other age groups (AOR = 2.76; 95% CI: 1.07–7.14; *p* = 0.037). This age group (25–34) can be considered as the most childbearing age that needs proper public health policies [10]. Pregnant women who had cats in their home, close contact with cats, lack of hand washing before meal, contact with garden soil, and drinking of tap water had significant association with seropositive pregnant women. These findings are in line with the routes of toxoplasma transmission such as drinking oocyst contaminated water, and contact with the soil. It is also indicated that domestic cats were the major sources of ecosystem contamination [17]. Furthermore, poor hygiene regarding to meat handling practices and lack of compliance with minimum meat hygiene and food safety standards may explain the increased level of toxoplasmosis in pregnant women in Somaliland [18].

In conclusion, toxoplasmosis in sheep and pregnant women is highly prevalent in Hargeisa, Somaliland. Environmental contamination by infected cats, contaminated tap water, and poor personal hygiene might have contributed to the widespread nature of toxoplasmosis in the area. The role of cats in the epidemiology of toxoplasmosis and the overall hygienic practices related to food handling should not be overlooked in Hargeisa, Somaliland.

## Author Contributions

**Abdiqani Mohamed Jama**: investigation, funding acquisition, formal analysis, resources, data curation, validation, methodology, writing – original draft, software, conceptualization. **Taddesse Yayeh**: supervision, writing – review and editing.

## Ethics Statement

The authors confirm that the ethical policies of the journal, as noted on the journal's author guidelines page, have been adhered to, and the appropriate ethical review committee approval (protocol number 01/IRB/2022) has been received. The College of Agriculture and Environmental Sciences guideline in Bahir Dar University for the Care and Use of Animals were followed.

## Conflicts of Interest

The authors declare no conflicts of interest.

## Data Availability

The data that support the findings of this study are available on request from the corresponding author. The data are not publicly available due to privacy or ethical restrictions.
